# Combination of Wogonin and Artesunate Exhibits Synergistic anti-Hepatocellular Carcinoma Effect by Increasing DNA-Damage-Inducible Alpha, Tumor Necrosis Factor α and Tumor Necrosis Factor Receptor-Associated Factor 3-mediated Apoptosis

**DOI:** 10.3389/fphar.2021.657080

**Published:** 2021-05-05

**Authors:** Minting Chen, Hsin Ling Wu, Tsz Sin Wong, Baisen Chen, Rui-Hong Gong, Hoi Leong Xavier Wong, Haitao Xiao, Zhaoxiang Bian, Hiu Yee Kwan

**Affiliations:** ^1^ Centre for Cancer and Inflammation Research, School of Chinese Medicine, Hong Kong Baptist University, Hong Kong, China; ^2^ School of Pharmaceutical Sciences, Health Science Center, Shenzhen University, Shenzhen, China

**Keywords:** artesunate, wogonin, hepatocellular carcinoma, GADD45a, GADD45, tumor necrosis factor-α, TRAF3

## Abstract

Hepatocellular carcinoma (HCC) is difficult to treat, and is the second leading cause of cancer-related death worldwide. This study aimed to examine whether combination of wogonin and artesunate exhibits synergistic anti-HCC effect. Our data show that the combination treatment exhibits synergistic effect in reducing HCC cell viability by increasing apoptosis as indicated by the elevated cleavage of caspase 8, 3 and PARP. Interestingly, PCR array and the subsequent studies indicate that the combination treatment significantly increases the expression of DNA-damage-inducible, alpha (GADD45A), tumor necrosis factor (TNFα) and TNF receptor-associated factor 3 (TRAF3). Knockdown of GADD45A, TNFα or TRAF3 abolishes the combination treatment-enhanced apoptosis and the synergistic effect in reducing HCC cell viability. In the HCC-bearing xenograft mouse models, although the combination treatment increases the activity of NFκB in the tumor tissues, it exhibits a more potent anti-HCC effect than the mono-treatment, which may due to the enhanced apoptosis as indicated by the increased expression of GADD45A, TNFα, TRAF3 and apoptotic markers. Our study clearly demonstrates that the combination of artesunate and wogonin exhibits synergistic anti-HCC effect, and support the further development of this combination as alternative therapeutics for HCC management.

## Introduction

Hepatocellular carcinoma (HCC) accounts for around 75–90% of all the primary liver cancer cases (Ringehan et al., 2017). It is the fifth most prevalent cancer (Lim and Torresi, 2021) and the second leading cause of cancer-related death worldwide (Lim and Torresi, 2021). One of the current clinical challenges for HCC management is that most of the HCC patients are diagnosed at an advanced stage along with concomitant complications. The main treatment for advanced HCC is the targeted therapy such as sorafenib. Sorafenib is a dual-target inhibitor targeting the serine-threonine kinase Raf and the receptor tyrosine kinases, vascular endothelial growth factor receptor (VEGF-R) and platelet-derived growth factor receptor (PDGF-R). Sorafenib exhibits significant effect in prolonging the overall survival of the HCC patients. However, these patients usually acquire resistance to sorafenib (Villanueva and Llovet, 2012). Indeed, HCC is one of the most difficult to cure cancers because of its high heterogeneity that attributes to the development of chemo-resistance, and hence poses great challenges to the management (Yang et al., 2019; Cabral et al., 2020).

Combination treatments have several advantages. It may exhibit synergistic effect that enhances the therapeutic efficacy, it may also reduce the likelihood of the acquired resistance in the patients (Palmer and Sorger, 2017). In Chinese medicine, a formula named *Haoqin Qingdan* Decoction is a commonly prescribed herbal medicine. It can be used to treat liver-related diseases such as jaundice hepatitis, acute cholecystitis and acute gallstone disease. In this formula, the constituent herbs *artemisia annua* and *Scutellaria baicalensis* are the main players that exert the therapeutic effects. Artemisinin and wogonin are one of the dominant bioactive compounds in *A. annua* and *S. baicalensis*, respectively.

Artemisinin has poor solubility in water and oil but only soluble in aprotic organic solvents. It is also unstable in the presence of both alkali and acid (Li and Zhou, 2010). Therefore, semisynthetic derivatives of artemisinin are synthesized, artesunate is one of these derivatives. The chemical property and the pharmacokinetic profile of artesunate make it more suitable as drug for disease treatments when compared to artemisinin (Morris et al., 2011). Furthermore, artesunate is water-soluble and can be applied by oral intake and intravenous or intramuscular injection. Indeed, artesunate is an approved frontline drug for severe malaria treatment. Experimental studies also suggest that artesunate has anti-HCC effects. For example., artesunate inhibits the proliferation of the HCC cells, induces apoptosis, downregulates the expressions of VEGF and reduces tumor vessel density (Hou et al., 2008) and tumor burden (Vandewynckel et al., 2014). Interestingly, artesunate significantly enhances the therapeutic effects of sorafenib in the treatment of HCC (Vandewynckel et al., 2014; Li et al., 2019).

Wogonin (5,7-dihydroxy-8-methoxyflavone) is a flavonoid-like chemical compound known to have anti-cancer effects, it suppresses the activity of matrix metalloproteinase-9, reduces the metastatic ability of HCC cells (Hong et al., 2018) and induces cell cycle arrest by activating glycogen synthase kinase-3β in HCC (Hong et al., 2020). Wogonin also enhances the therapeutic effects of sorafenib in the treatment of HCC by increasing apoptosis and inhibiting autophagy (Rong et al., 2017).

Given both artesunate and wogonin have anti-HCC effects, we aimed to examine whether combination of artesunate and wogonin will have synergistic effect in HCC treatment and whether the combination treatment will have a more potent anti-HCC effect than sorafenib.

## Materials and Methods

### Materials

Wogonin and Poerce(R) BCA Protein Assay Kit were purchased from Sigma-Aldrich (Munich, Germany). Artesunate was purchased from Sigma-Aldrich. Antibodies against cleaved-poly-(ADP-ribose) polymerase (cleaved-PARP), cleaved-caspase 8, cleaved-caspase 3, cleaved-caspase 9, full length caspase 12, cleaved-caspase 12, full length caspase 7, cleaved-caspase 7, and *ß*-Actin were purchased from Cell Signaling Technology. Antibody against full length caspase 6, cleaved-caspase 6, full length caspase 10, cleaved-caspase 10, and full length caspase 4 were purchased from Abcam (Cambridge, United Kingdom). Mouse anti-rabbit IgG-HRP secondary antibody was purchased from San Cruz Biotechnology (Santa Cruz, United States of America).

### Cell Culture

HepG2 and Hep3B cells were purchased from American Type Culture Collection (ATCC; Rockville, MD, United States). Culture medium and FBS were obtained from Gibco (Thermo Fisher Scientific, United States). Cells were cultured in DMEM supplemented with 10% FBS in a humidified atmosphere containing 5% CO_2_ and 95% air at 37°C.

### MTT Assay

The viability of HepG2 and Hep3B cells after the treatments were assessed by MTT [3-(4,5-dimethylthiazol-2-yl)-2,5-diphenyltetrazolium bromide] assay. Cells (2,000 per well) were seeded in 96-well plates 24 h prior to treatment. The synergistic effect of artesunate and wogonin was determined by combining different concentrations of the compounds and calculated for the combination index (CI) with CompuSyn software 2.0 according to the software’s instruction.

### siRNA Transfection

Transient transfections of siRNA was accomplished using Lipofectamine RNAiMAX (Invitrogen) transfecting reagent according to manufacturer’s instructions. Briefly, HepG2 cells were seeded in 12-well plates and transfected using 20 μM siRNA and 3 μL Lipofectamine RNAiMAX for 24 h.

### Annexin V/PI Staining for Cell Apoptosis Analysis

Apoptosis of the HCC cells treated with artesunate, wogonin, or the combination of both were analyzed by flow cytometry following the protocol of FITC Annexin V Apoptosis Detection Kit (BD Biosciences). Briefly, HepG2, Hep3B, si-GADD45A HepG2, si-TNF HepG2 and si-TRAF3 HepG2 cells were separately treated with artesunate, wogonin, or combination of both for 48 h. Then the cells were harvested and washed with PBS and stained with FITC Annexin V and propidium iodide (PI) in the dark for 30 min. The apoptotic cell distribution was examined by BD FACS Calibur flow cytometer and analyzed by BD FACSVia Research Loader Software.

### Western Blot Analysis

Cells were seeded in 6 well plates, and then treated with artesunate, wogonin, or combination of both for 48 h. After that, whole cell lysates were obtained by suspending the cells in RIPA reagent, followed by centrifugation at 15,000 rpm for 15 min at 4 °C. The protein samples of the tumor tissues were also prepared with RIPA reagent followed by centrifugation. Total protein concentration was measured by Pierce(R) BCA Protein Assay Kit, and 10–30 μg of protein was separated on 8–12% SDS-PAGE. The PVDF membrane carrying transferred proteins was incubated at 4°C overnight with the corresponding primary antibody at 1:1,000 ratio. The immune-reactive proteins were detected by enhanced chemiluminescence (ECL) detection system using X-ray film and ECL reagent.

### Real-Time Polymerase Chain Reaction Analysis

Total RNA was extracted with TRIzol reagent (Invitrogen), and cDNAs were subsequently prepared by reverse transcription. Human Apoptosis RT Profiler PCR Array experiment was performed following the instruction of RT^2^ Profiler PCR Array kit (QIAGEN). Real-time polymerase chain reaction (PCR) was performed using the TB Green PCR Master mix (TaKaRa) with 2 μL cDNA in a final volume of 20 μL and the following primers at a final concentration of 1,000 nM. Primers for GADD45A were 5′-GTT​TTG​CTG​CGA​GAA​CGA​C-3’ (forward) and 5′-GAA​CCC​ATT​GAT​CCA​TGT​AG-3’ (reverse). Primers for TRAF3 were 5′-CTC​ACA​AGT​GCA​GCG​TCC​AG-3’ (forward) and 5′-GCT​CCA​CTC​CTT​CAG​CAG​GTT-3’ (reverse). Primers for TNF were 5′-CTC​TTC​TGC​CTG​CTG​CAC​TT-3’ (forward) and 5′-GGC​TAC​AGG​CTT​GTC​ACT​C-3’ (reverse). Primers for HRK were 5′-GGC​AGG​CGG​AAC​TTG​TAG​GAA​C-3’ (forward) and 5′-TCC​AGG​CGC​TGT​CTT​TAC​TCT​CC-3’ (reverse). Primers for TNFRSF11 B were 5′-TTG​GTC​TCC​TGC​TAA​CTC​A-3’ (forward) and 5′-GAA​GAA​TGC​CTC​CTC​ACA​C-3’ (reverse). Primers for PYCARD were 5′-AAC​CCA​AGC​AAG​ATG​CGG​AAG-3’ (forward) and 5′-TTA​GGG​CCT​GGA​GGA​GCA​AG-3’ (reverse). Amplifcation of HRK, GADD45A, PYCARD, TNF, TNFRSF11B, TRAF3 cDNAs was performed using the ViiA 7 Real-Time PCR System (Thermo Fisher Scientific). The cycling conditions comprised a denaturation step for 15 min at 95°C, followed by 40 cycles of denaturation (95°C for 15 s), annealing (59°C for 20 s), and extension (72°C for 15 s). After amplifcation, a melting curve analysis was performed with denaturation at 95°C for 5 s, then continuous fluorescence measurement was made from 70 to 95°C at 0.1°C/s. Each sample was amplified in duplicate.

### Xenograft Mouse Model

Six-week old male nude mice were purchased from the Laboratory Animal Services Center, The Chinese University of Hong Kong. Mice were kept at room temperature 23 ± 2°C with an alternating 12 h light-dark cycle and were allowed free access to food and water. All of the experimental protocols were carried out with the approval of the Ethical Committee at the Hong Kong Baptist University. HepG2 cells (1× 10^6^ cells per mouse) were suspended in PBS and inoculated subcutaneously into the left flank of each mouse. The tumor growth was monitored every day. When tumors were grown to ∼100 mm^3^, mice were randomly divided into six groups, each group with 3-5 mice, the treatments were as follows: 1) vehicle control group 1 (i.p. 0.5% NaHCO_3_ and 0.2% NaOH in saline), 2) artesunate (ATN) group (i.p. 60 mg/kg of ATN), 3) wogonin (WOG) group (i.p. 60 mg/kg of WOG), 4) ATN combined with WOG group (i.p. 60 mg/kg of ATN and 60 mg/kg of WOG), 5) vehicle control group 2 for sorafenib (i.p. 1% DMSO in soybean oil) 6) sorafenib group (daily i. p. 10 mg/kg of sorafenib). The tumors were measured with calipers, and tumor volumes were calculated by the following formula: a^2^×*b*×0.5, where “a” is the smallest diameter and “b” is the diameter perpendicular to “a”. At the end of the experiment, the mice were sacrificed, the tumor tissues were dissected and measured.

### H&E Staining and Protein Extraction From Tumors

Tumors were fixed in 4% neutral buffered paraformaldehyde at 4°C for 24 h. Samples were then embedded in paraffin, sectioned and treated in the following steps for H&E staining: hematoxylin for 10 min, 1% acid–ethanol for 30 s, 1% ammonia water for 30 s, and eosin for 10 s.

### Statistical Analysis

The data shown in the study were obtained in at least three independent experiments and results are expressed as mean ± standard error. Statistical comparison between control and treatments was carried out using one-way ANOVA with GraphPad Prism 8.0 software. Data are taken as significant when *p*<0.05. Combination Indexwas calculated by CompuSyn software 2.0 according to the software’s instruction.

## Results

### Combination of Artesunate and Wogonin Exhibits Synergistic Effect in Reducing Hepatocellular Carcinoma Cell Viability

**FIGURE 1:**
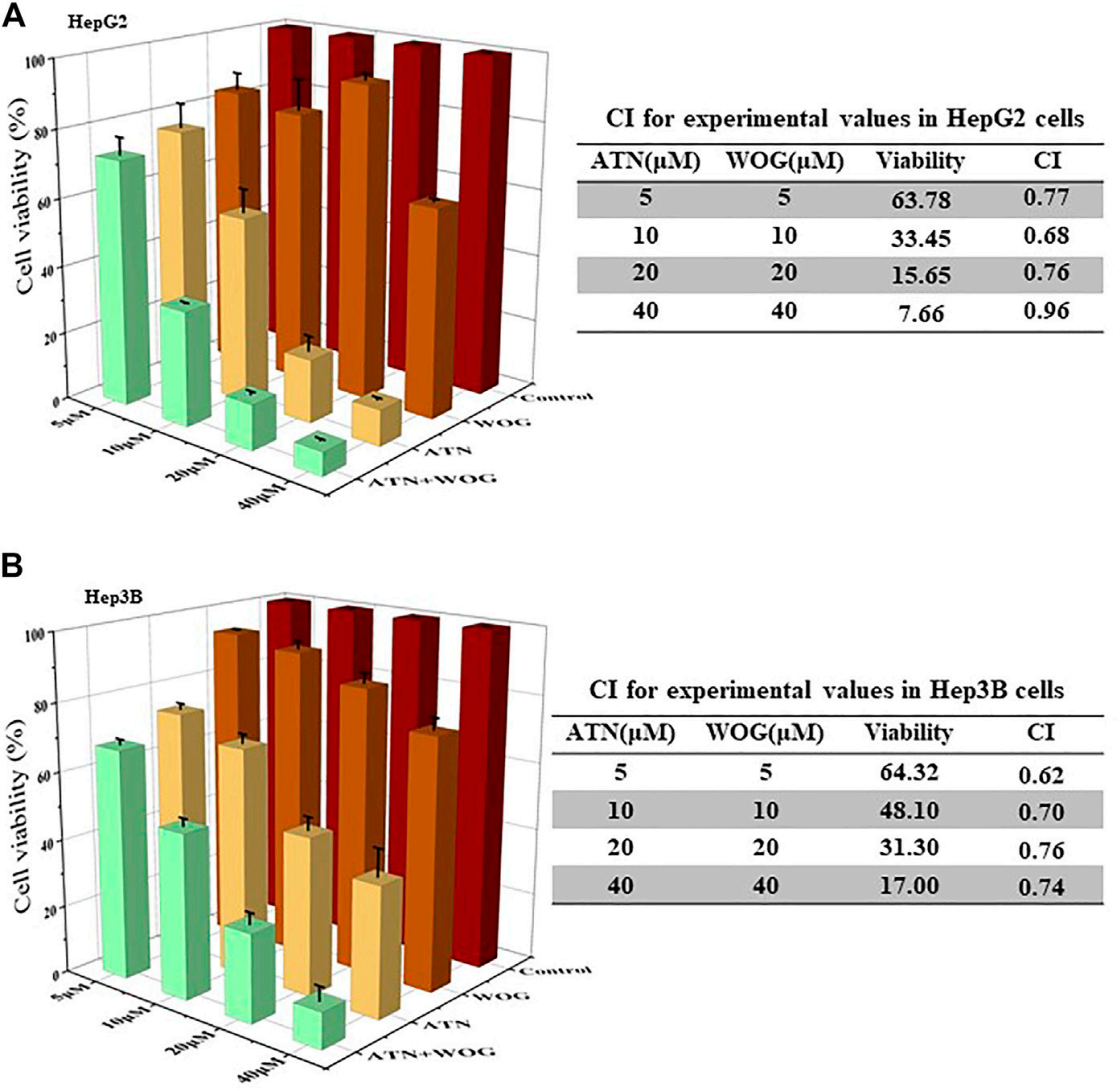
Combination of artesunate and wogonin exhibits synergistic effect in inhibiting HCC cell proliferation. **(A)**. HepG2 and **(B).** Hep3B cells were treated with different doses of ATN (5, 10, 20, 40 μM), WOG (5, 10, 20, 40 μM), or combinations of both. Cell viabilities were measured by MTT assay after 72 h treatment. ATN, artesunate; WOG, wogonin. Shown is mean ± SE, *n* = 3 independent experiments.

HCC cells HepG2 and Hep3B were used for the study. The synergy of artesunate and wogonin was determined by combination index (CI) based on the median-effect equation, derived from the mass-action law principle. The resulting CI theorem offers quantitative definition for additive effect (CI = 1), synergism (CI < 1), and antagonism (CI > 1) in the drug combinations (Chao, 2010). Figure 1A showed that the combination treatment exhibited synergistic effect in reducing HepG2 cell viability as CI < 1. Similar results of CI < 1 under the combination treatment were also observed in Hep3B cells (Figure 1B), Huh7 cells (Supplementary Figure S1A) and SMMC7721 cells (Supplementary Figure S1B).

Combination of artesunate and wogonin significantly increases apoptosis in the HCC cells, the treatment does not affect autophagy, cell cycle arrest and angiogenesis.

Next, we investigated the mechanisms of action underlying the synergistic effect of the combination treatment. Since artesunate and wogonin induce autophagy in colorectal cancer cells (Jiang et al., 2018) and nasopharygeal carcinoma cells (Chow et al., 2012), respectively, we first examined whether the treatments affected autophagy in HCC cells. p62 is an autophagy marker. p62 protein recognizes toxic cellular waste, which then initiates autophagy. Therefore, accumulation of p62 indicates an inhibition of the autophagy. Besides, during autophagy, the microtubule-associated protein 1A/1B-light chain 3 (LC3) will be conjugated to phosphatidylethanolamine to form LC3-phosphatidylethanolamine conjugate (LC3-II), which is then recruited to the autophagosomal membrane. As shown in Supplementary Figure S2, neither the mono-treatments nor the combination treatment affect the expression of p62, LC3-I and LC3-II in the HepG2 cells after 24, 48 or 72 h treatments, suggesting that autophagy may not mediate the synergy of the combination treatment.

We also examined whether the combination treatment affected cell cycles in HCC cells. During cell cycle, cyclin D1 is responsible for the cell cycle progression at G1S phase, where it initiates DNA synthesis. While CDK2 is a key regulator of G1-S cell cycle progression. Upon the phosphorylation at threonine 160 residue by CDK-activating kinase, CDK2 will be activated. p21 (CDKN1A) and p27 (CDKN1B) are the two G1-checkpoint CDK inhibitors. Supplementary Figure S3A showed that the combination treatment reduced the expression of p21 and p27, and increased the phosphorylation of CDK2 at 72 h; however, it did not significantly affect the expression of cyclin D1. Since downregulation of p21 and p27 could affect cell cycle through cyclin E, we performed flow cytometry analysis to examine the cell cycles of the HCC cells after treatments. As shown in Supplementary Figure S3B–S3G, the combination treatments did not induce cell cycle arrest in the HepG2 cells. Instead, it reduced the cell number at G1/G0 phase after 48 and 72 h treatments (Supplementary Figure S3F–S3G), which may not underline the reduced cell viability after the treatments.

Besides, we also found that the treatments did not affect the expression of angiogenic markers in the HCC cells such as VEGF (Supplementary Figure S4) that is an important signaling molecule in vasculogenesis and angiogenesis, and CD31 (Supplementary Figure S4) that promotes HCC metastasis by inducing epithelial-mesenchymal transition.

**FIGURE 2:**
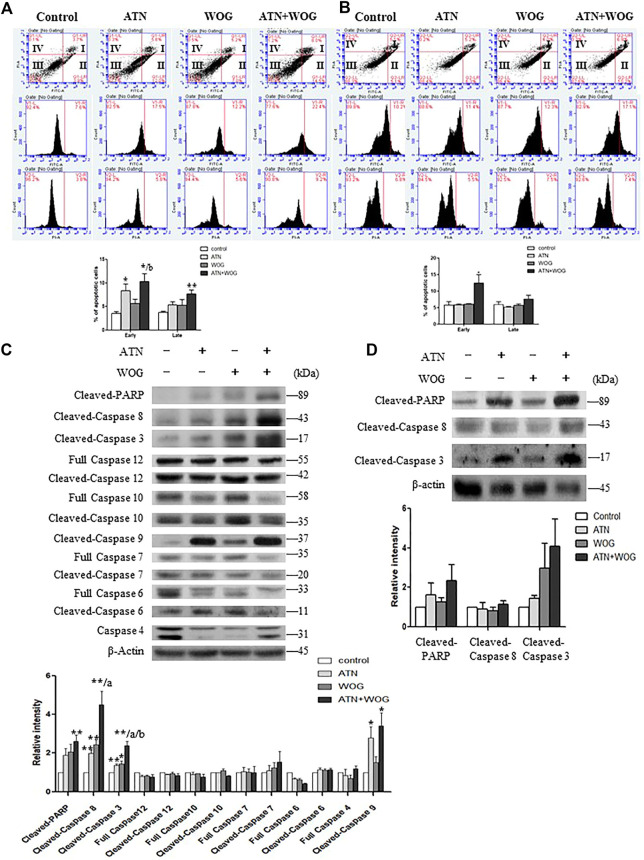
Combination of artesunate and wogonin significantly increases apoptosis in the HCC cells. **(A)**. HepG2 cells and **(B)**. Hep3B cells treated with ATN (10 μM), WOG (10 μM) or combination of both for 48 h were stained with Annexin V/PI. Vehicle served as control. Flow cytometry analysis of the percentages of early and late apoptosis in these cells **(bottom panel)**. **(C).** Expression of cleaved-PARP, cleaved-caspase 8, cleaved-caspase 3, full length caspase 12, cleaved-caspase 12, full length caspase 10, cleaved-caspase 10, cleaved-caspase 9, full length caspase 7, cleaved-caspase 7, full length caspase 6, cleaved-caspase 6 and full length caspase 4, and quantitative analysis of the protein expressions, of the HepG2 cells treated with ATN (10 μM), WOG (10 μM) or the combination of both for 48 h. **(D)** Expression of cleaved-PARP, cleaved-caspase 8 and cleaved-caspase 3, and quantitative analysis of the protein expressions, of the Hep3B cells treated with ATN (10 μM), WOG (10 μM) or combination of both for 48 h. Shown is mean ± SE, n = 3 independent experiments. 1*p < 0.05, **p < 0.01, compared with control; a<0.05, aa<0.01 compared with ATN; b < 0.05, compared with WOG. ATN, artesunate; WOG, wogonin; PI, propidium iodide; Cleaved-PARP, cleaved-poly-(ADP-ribose) polymerase.

Interestingly, we found that the combination treatment significantly increased the percentages of early and late apoptotic cells as examined by Annexin V and propidium iodide staining assessed by flow cytometry (Figures 2A,B). Among all the initiator caspases, the combination treatment significantly enhanced the cleavage of caspase 8 in the HCC cells (Figures 2C,D). The cleavage and activation of caspase 8 implies an activation of the extrinsic apoptotic pathway. The extrinsic pathway is known to be initiated by the activation of membrane death receptors and formation of death-inducing signaling complex (DISC), in which caspase 8 is activated and promotes apoptosis by activating the effector caspases such as caspase 3 which in turns cleaves poly (ADP-ribose) polymerase (PARP). Indeed, our data showed that the combination treatment significantly increased the cleavage of caspase 3 and PARP in the HCC cells (Figures 2C,D). These data strongly suggest that apoptosis in the HCC cells is significantly increased after the combination treatment when comparing to the mono-treatments.

### Combination of Artesunate and Wogonin Changes the Profiles of the Apoptosis-Related Genes in Hepatocellular Carcinoma Cells

**FIGURE 3:**
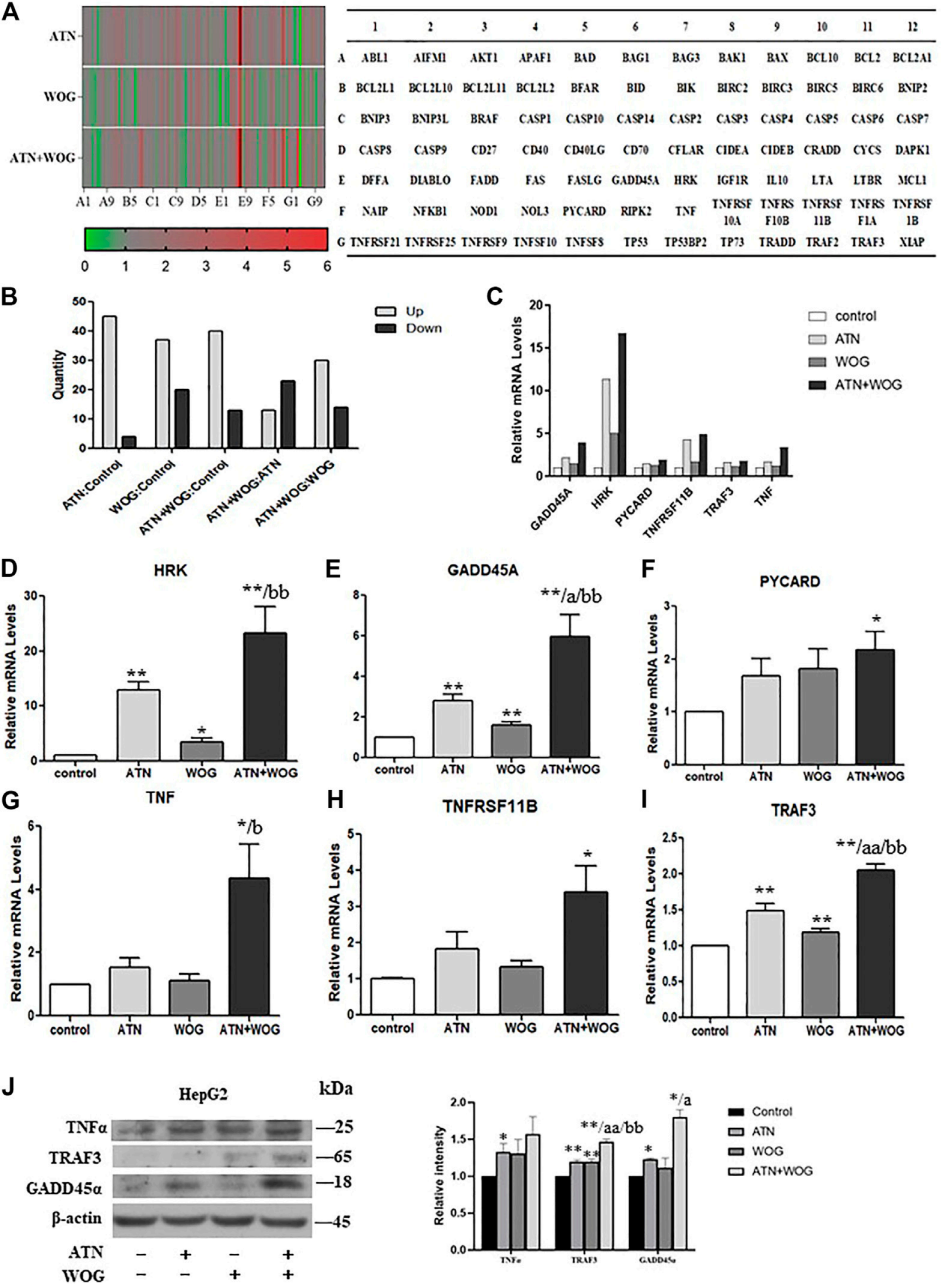
Combination of artesunate and wogonin changes the profiles of the apoptosis-related genes in HCC cells. **(A).** Heat map of the PCR array showing the expression levels of the apoptosis-related genes in the HepG2 cells treated with ATN (10 μM), WOG (10 μM) or the combination of both for 48 h. Red color demonstrated an increased, and green color demonstrated a reduced expression of the genes. A table **(right panel)** showing the genes in the PCR array in the corresponding position (gene A1 in the figure corresponds to the gene ABL1 in the table). **(B).** Analysis of the number of altered genes between different treatment groups. **(C).** The mRNA expression level of GADD45A, HRK, PYCARD, TNFRSF11B, TRAF3 and TNF in the HepG2 cells treated with ATN (10 μM), WOG (10 μM) or the combination of both for 48 h. qPCR showing the relative mRNA levels of **(D)**. HRK, **(E)**. GADD45A, **(F)**. PYCARD, **(G)**. TNF, **(H)**. TNFRSF11 B and **(I)**. TRAF3 in HepG2 cells treated with ATN (10 μM), WOG (10 μM) or the combination of both for 48 h. **(J).** Protein expression of TNFα, TRAF3 and GADD45α, and quantitative analysis of the protein expressions, of the HepG2 cells treated with ATN (10 μM), WOG (10 μM) or the combination of both for 48 h. Shown is mean ± SE, n = 3 independent experiments. *p < 0.05, **p < 0.01, compared with control; a<0.05, aa<0.01 compared with ATN; b < 0.05, bb < 0.01, compared with WOG. ATN, artesunate; WOG, wogonin; GADD45A, growth arrest and DNA-damage-inducible alpha; HRK, harakiri; PYCARD, apoptosis-associated speck-like protein containing a caspase recruitment domain; TNFRSF11B, tumor necrosis factor receptor superfamily member 11b; TRAF3, TNF receptor-associated factor 3; TNF, tumor necrosis factor.

We next performed PCR array to investigate which apoptosis-related genes were affected by the combination treatment. A total of 84 apoptosis-related genes were tested in the array. The results were shown in the heat map (Figure 3A), the numbers of up- or down-regulated genes were presented in Figure 3B. The results showed that, compared to control, artesunate treatment upregulated 45 genes and downregulated 4 genes; wogonin treatment upregulated 37 genes and downregulated 20 genes; and the combination treatment upregulated 40 genes and downregulated 13 genes. Compared to artesunate treatment, the combination treatment upregulated 13 genes and downregulated 23 genes. Compared to wogonin treatment, the combination treatment upregulated 30 genes and downregulated 14 genes. These genes were shown in Supplementary Table S1.

Among all these apoptosis-related genes, six of them were significantly increased under the combination treatment when compared to the mono-treatments, they were harakiri BCL2 interacting protein (HRK), growth arrest and DNA-damage-inducible, alpha (GADD45A), PYD and CARD domain (PYCARD), tumor necrosis factor (TNFα), tumor necrosis factor receptor superfamily, member 11 b (TNFRSF11 B) and TNF receptor-associated factor 3 (TRAF3) (Figure 3C). The elevated mRNA levels of these genes were validated by qPCR (Figures 3D–I). However, only the protein levels of TNFα, TRAF3 and GADD45α were significantly increased after the combination treatments (Figure 3J).

### The Combination Treatment-Enhanced Apoptosis Is Mediated by Tumor Necrosis Factor α, TRAF3 and GADD45α in Hepatocellular Carcinoma Cells

**FIGURE 4:**
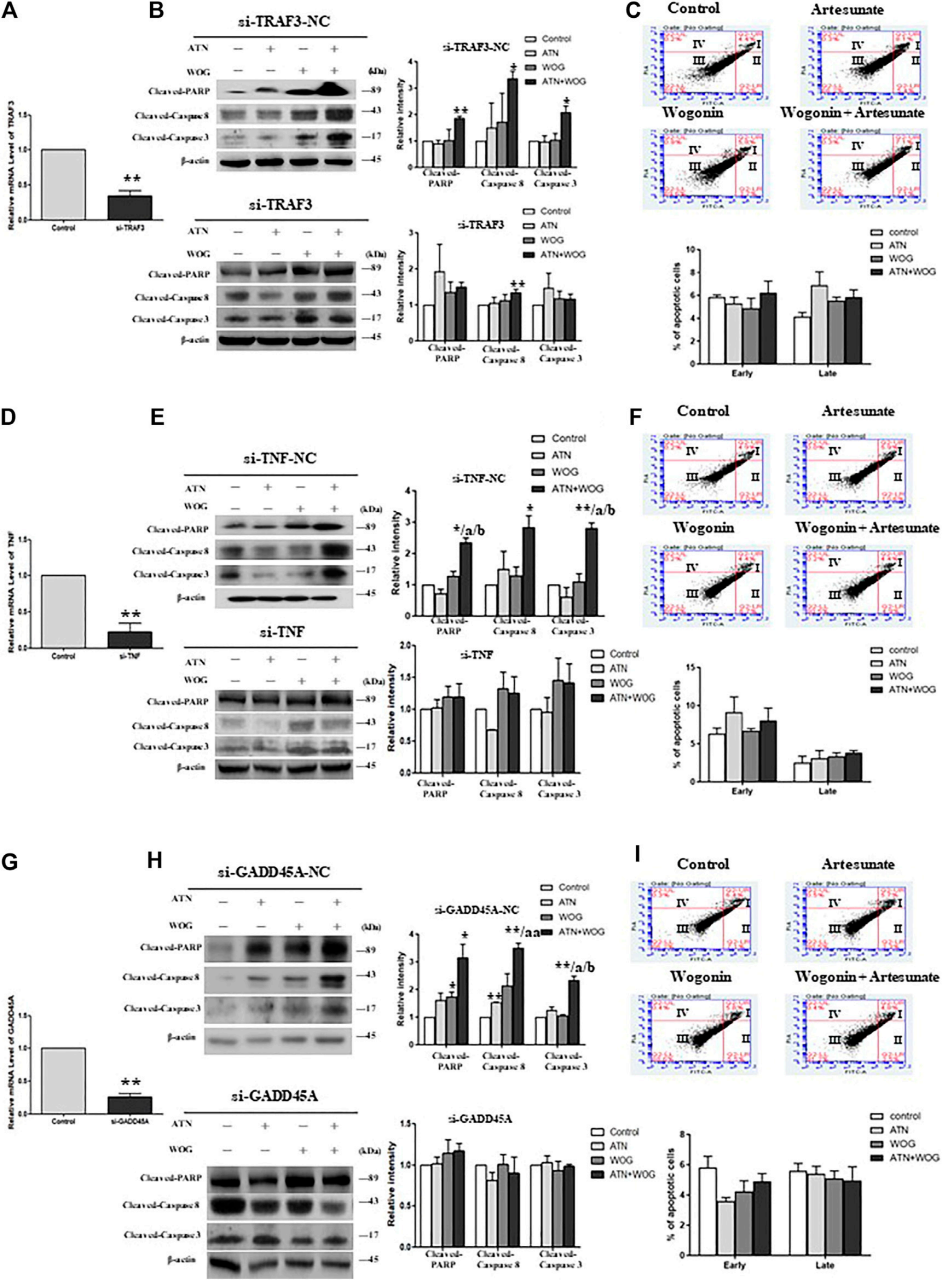
The combination treatment-enhanced apoptosis is mediated by TNFα, TRAF3 and GADD45α in HCC cells. Relative mRNA level of **(A)**. TRAF3, **(D)**. TNFα, and **(G).** GADD45A in HepG2 cells after the respective siRNA-mediated knockdown of the gene, negative control (NC)-siRNA served as control. Protein expression levels of cleaved-PARP, cleaved-caspase 8 and cleaved-caspase 3 in **(B)**. TRAF3-knockdown cells (si-TRAF3-HepG2) and control cells (si-TRAF3-NC), **(E)**. TNFα-knockdown cells (si-TNFα-HepG2) and control cells (si-TNF-NC), **(H)**. GADD45a-knockdown cells (si-GADD45A-HepG2) and control cells (si-GADD45A-NC), after the treatment with ATN (10 μM), WOG (10 μM) or the combination of both for 48 h. Flow cytometry analysis of the percentage level of early and late apoptosis in **(C)**. si-TRAF3-HepG2, **(F)**. si-TNFα-HepG2 and **(I)**. si-GADD45A-HepG2 cells after the treatment with ATN (10 μM), WOG (10 μM) or the combination of both for 48 h. Shown is mean ± SE, n = 3 independent experiments. **p < 0.01 compared to control, a<0.05, aa<0.01 compared with ATN; b < 0.05 compared with WOG. ATN, artesunate; WOG, wogonin; TRAF3, TNF receptor-associated factor 3; TNF, tumor necrosis factor; GADD45A, growth arrest and DNA-damage-inducible alpha; PI, propidium iodide; Cleaved-PARP, cleaved-poly-(ADP-ribose) polymerase.

To investigate whether TNFα, TRAF3 and GADD45α mediated the enhanced apoptosis under the combination treatments, we used the respective siRNA to mediate the gene knockdown in HCC cells (Figures 4A,D,G). In the TNFα-knockdown, TRAF3-knockdown and GADD45A-knockdown cells, mono-treatments and the combination treatment failed to increase the cleavage of caspase 8, 3 and PARP (Figures 4B,E,H); and did not significantly increase the percentages of the apoptotic cells (Figures 4C,F,I). These data suggest that TNFα, TRAF3 and GADD45α are involved in the apoptosis under these treatments.

We also used siRNA to mediate the knockdown of the gene encoding HRK, PYCARD or TNFRSF11B in the cells to examine whether HRK, PYCARD and TNFRSF11B were also involved in the combination treatment-enhanced apoptosis. As shown in the Supplementary Figure S5, after HRK or TNFRSF11B or PYCARD was knockdown, the combination treatment could still significantly enhance the expressions of the apoptotic markers when compared to the control or the mono-treatments. These data suggest that HRK, PYCARD and TNFRSF11 B are less likely involved, or they are not playing the dominant roles, in the combination treatment-enhanced apoptosis in the HCC cells.

### Knockdown of TNFα, TRAF3 and GADD45α Abolish the Synergistic Effect of the Combination Treatment in Reducing the Hepatocellular Carcinoma Cell Viability

**FIGURE 5:**
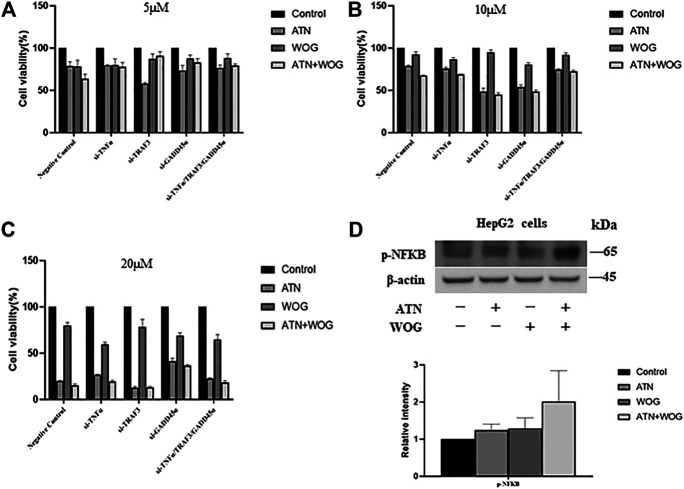
Knockdown of TNFα, TRAF3 or GADD45α abolishes the synergistic effect of the combination treatment in reducing the HCC cell viability. Cell viability of TRAF3-knockdown cells (si-TRAF3-HepG2), TNFα-knockdown cells (si-TNFα-HepG2) and GADD45a-knockdown cells (si-GADD45A-HepG2) after treating with **(A)**. ATN (5 μM), WOG (5 μM) or the combination of both for 48 h; or treated with **(B).** ATN (10 μM), WOG (10 μM) or the combination of both; or **(C)**. ATN (20 μM), WOG (20 μM) or the combination of both for 48 h. **(D).** Expressions of phosphorylated NFκB (*p*-NFκB) in HepG2 cells after treating with ATN (10 μM), WOG (10 μM) or the combination of both for 48 h. Shown is mean ± SE, *n* = 3 independent experiments.

Our data strongly suggest that the combination treatment significantly increases apoptosis and has synergistic effect in reducing HCC cell viability, we next examine whether the synergistic effect is due to the enhanced apoptosis. Since knockdown of TNFα, TRAF3 and GADD45α in the HCC cells abolished the increased apoptosis under the combination treatments (Figure 4), we used these cells as models to examine whether the combination treatment could still have synergistic effect. As shown in Figures 5A–C, the combination treatments at 5–20 µM did not have synergistic effect in inhibiting the cell viability with these cell models. The result suggests that the synergistic effect of the combination treatment is due to the enhanced apoptosis mediated by TNFα, TRAF3 and GADD45α in the HCC cells.

Other reports suggests that TNFα has dual roles (Wang and Lin, 2008). TNF *a* is known to induce apoptosis in a variety of cell types by forming a complex that consists of caspase 8, fas-associating protein with death domain (FADD), tumor necrosis factor receptor type 1-associated DEATH domain protein (TRADD) and receptor interacting protein (RIP) (Wang, 2001). However, TNFα also induces NFκB activation that promotes cancer growth (Karin et al., 2004). Since the combination treatments increased TNFα expressions, we examined whether NFκB was also activated in the HCC cells. As shown in Figure 5D, neither the mono-treatment nor the combination treatment significantly increased the phosphorylation of NFκB in the HepG2 cells, suggesting that the elevation of TNFα does not significantly affect the activity of NFκB.

### The Combination Treatment Significantly Reduces Tumor Growth and Exhibits a More Potent anti-Hepatocellular Carcinoma Effect Than Sorafenib *in vivo*

**FIGURE 6:**
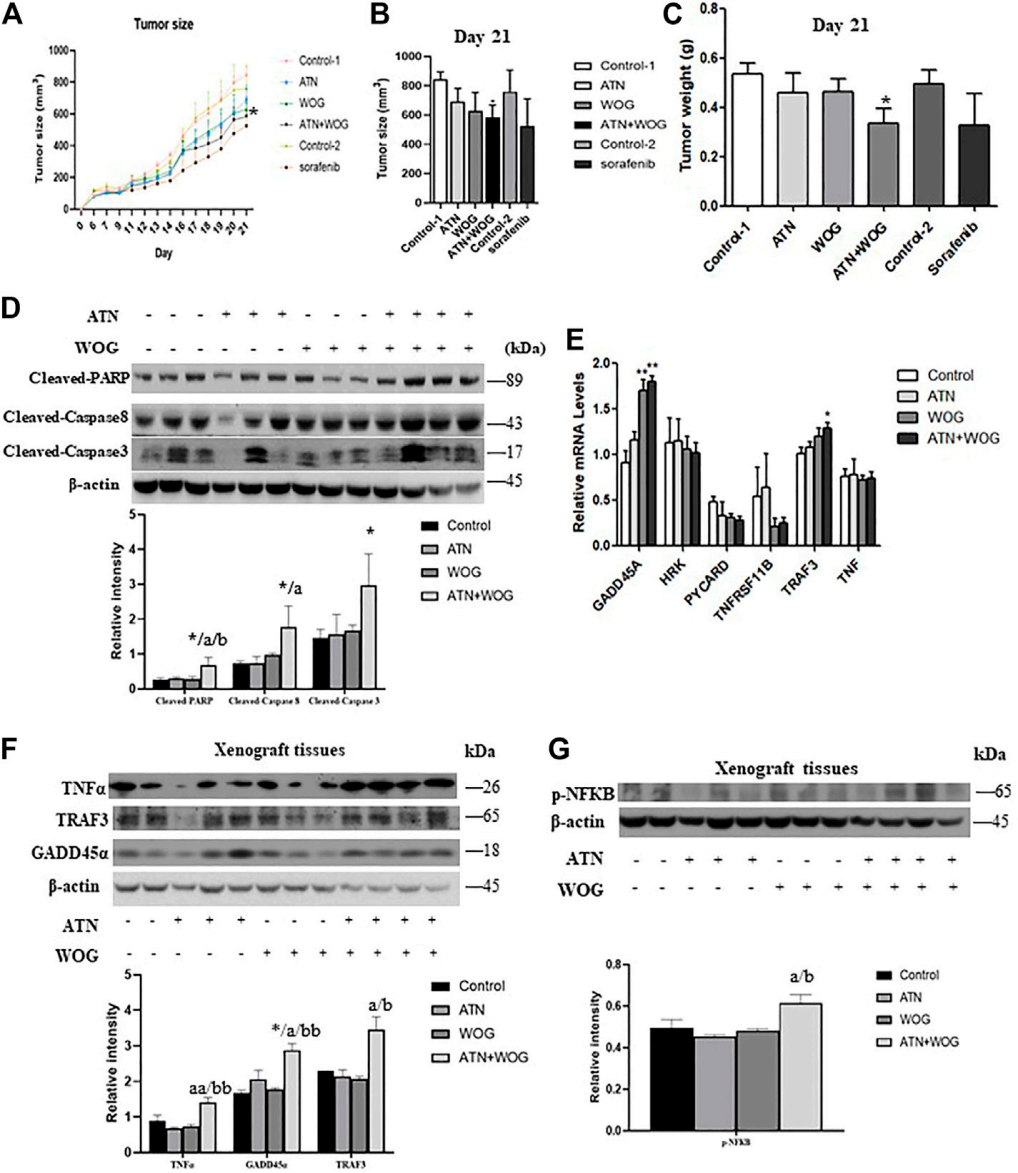
The combination treatment significantly reduces tumor growth and exhibits a more potent anti-HCC effect than sorafenib *in vivo.* Six-week old male nude mice were inoculated subcutaneously with injections of HepG2 cells and randomly divided into 6 groups: (1) vehicle control (control 1) (i.p. 0.5% NaHCO_3_ and 0.2% NaOH in saline), (2) artesunate (ATN) group (i.p. 60 mg/kg of ATN), (3) wogonin (WOG) group (i.p. 60 mg/kg of WOG), (4) ATN combined with WOG group (i.p. 60 mg/kg of ATN and 60 mg/kg of WOG), (5) vehicle control group for sorafenib (control 2) (i.p. 1% DMSO in soybean oil), (6) sorafenib group (daily i. p. 10 mg/kg of sorafenib). **(A).** Tumor size during the course of treatment, **(B)**. tumor size on day 21, and **(C)**. tumor weight of the mice after the treatments. **(D).** Expressions of cleaved PARP, caspase 8 and 3, and quantitative analysis of the expressions, in the tumor tissues after treatments. **(E).** Relative mRNA and **(F).** protein expressions and quantification of TNFα, TRAF3 and GADD45α in the tumor tissues after treatments. **(G)**. Protein expression and quantification of phosphorylated NFκB (*p*-NFκB) in the tumor tissues after treatments. Shown is mean ± SE, *n* = 3-5 mice in each group. *p < 0.05, **p < 0.01 compared with control group, a<0.05, aa<0.01 compared with ATN group; b < 0.05, bb < 0.01 compared with WOG group. ATN, artesunate; WOG, wogonin; GADD45A, growth arrest and DNA-damage-inducible alpha; HRK, Harakiri; PYCARD, apoptosis-associated speck-like protein containing a caspase recruitment domain; TNFRSF11B, tumor necrosis factor receptor superfamily member 11b; TRAF3, TNF receptor-associated factor 3; TNF, tumor necrosis factor; PI, propidium iodide; Cleaved-PARP, cleaved-poly-(ADP-ribose) polymerase.

Next, we examined whether the combination treatment exhibited more potent anti-HCC effect than the mono-treatments in xenograft mouse model. Since epidemiology studies show that male have higher incidences of HCC and poorer responses to treatments than the female (Greten, 2019; Natri et al., 2019), we used male nude mice to establish the xenograft mouse model for the study. The nude mice were subcutaneously injected the HepG2 cells and received the treatments when the tumors were grown to around 100 mm^3^ in size. We found that the mono-treatments did not significantly reduce the tumor size (Figures 6A,B) and tumor weight (Figure 6C) at the dosage of 60 mg/kg for artesunate or 60 mg/kg for wogonin. However, the combination of artesunate (60 mg/kg) and wogonin (60 mg/kg) significantly reduced the tumor size and tumor weight in these mice (Figures 6A–C), suggesting that the combination treatment exhibits a more potent anti-HCC effect than the mono-treatments. All the treatments did not have apparent toxicity to the mice as indicated by the body weight of the mice (Supplementary Figure S6A) and the histological examination of the major organs (Supplementary Figure S6B).

We also examined whether the combination treatment induced more apoptosis in the tumor tissues than the mono-treatments did. In parallel with the results in the *in vitro* studies, combination treatment, but not mono-treatments, significantly increased the cleavage of caspase 8, 3 and PARP (Figure 6D). Besides, the combination treatment also significantly increased the mRNA levels of TRAF3 and GADD45A (Figure 6E) and the protein levels of TNFα, TRAF3 and GADD45A (Figure 6F) in the tumor tissues of these mice. However, the combination treatment slightly but significantly enhanced the activity of NFκB in the tumor tissues (Figure 6G). Taken together, these data suggest that the potent anti-HCC effect of the combination treatment is, at least in part, due to the enhanced apoptosis in the tumor.

Besides, we also included sorafenib in the study. The standard starting dosage of sorafenib (NEXAVAR) for patients is 800 mg/day (Reiss et al., 2017). However, this standard dosage may result in grade 2 to 4 adverse reactions such as cardiovascular events, hemorrhage, hypertension, gastrointestinal perforation, QT prolongation, severe drug-induced liver injury and non-hematological toxicities. Therefore, in the study, we reduced the standard dosage and used the corresponding mouse dosage of 10 mg/kg/day for the treatment. The results showed that the sorafenib treatment did not have apparent toxicity to the mice as it did not significantly affect the body weight of the mice (Supplementary Figure S5A). However, unlike the combination treatment, sorafenib treatment failed to significantly reduce the tumor size and tumor weight when compared to its corresponding control treatment (Figures 6A–C). These results suggest that the combination treatment exhibits more potent anti-HCC effect than sorafenib in these mouse models at their safe dosages.

## Discussion

Our study suggests that combination of artesunate and wogonin significantly increases apoptosis in HCC, which is mediated by the elevated expression of TNFα, TRAF3 and GADD45A. After TNFα, TRAF3 or GADD45A is knockdown in the HCC cells, both mono-treatments and combination treatment failed to induce apoptosis; and the combination treatment failed to exhibit its synergistic effect in inhibiting the cancer growth. Since these treatments do not significantly affect autophagy, cell cycle and angiogenesis, we suggest that autophagy, cell cycle arrest and inhibition of angiogenesis do not underlie the synergy of the combination treatment.

TNFα, TRAF3 and GADD45A are all apoptosis-related signaling molecules. TNFα is known to induce apoptosis, the TNF receptor (TNF-R1) is a classical death receptor which forms complex with TRADD, FADD and RIP, and is associated to pro-caspase 8. The activated caspase 8 subsequently cleaves an effector caspase, such as caspase 3, which induces apoptosis. Our results clearly demonstrate that cleavage of caspase 8 is enhanced after the combination treatment, which further suggests the activation of this TNFα/caspase 8 extrinsic apoptotic pathway. Indeed, TNFα also increases the production of reactive oxygen species which lead to cell death by activating c-Jun N-terminal kinase (JNK) (Kim et al., 2010). Besides, TNFα also induces extracellular Ca^2+^ influx into cytoplasm through transient receptor potential channel in HCC cells (Zhu et al., 2018). The elevated extracellular Ca^2+^ influx accelerates TNFα-induced extrinsic apoptosis by activating the calpain/IAP/caspase3 pathway (Zhu et al., 2018). TRAF3 has been reported to induce apoptosis in HCC (Ding et al., 2020), which involves the activation of the JNK/activator protein-1 (AP-1) signaling pathway (Georgopoulos et al., 2006). Whereas GADD45A induces apoptosis either *via* p53-dependent or p53-independent pathway. In the p53-dependent pathway, GADD45A is activated by the mitogen-activated protein kinase (MAPK) signaling pathway under stress, which in turn activates p38 and JNK (Liebermann et al., 2011). In the p53-independent pathways, GADD45A induces Bim dissociation that leads to apoptosis (Tong et al., 2005). In our study, we could not separately evaluate the contribution TNFα, TRAF3 and GADD45A in the combination treatment-enhanced apoptosis because knockdown of either TNFα, TRAF3 or GADD45A can completely abolish the treatment-induced apoptosis and the synergy in inhibiting cancer cell growth. This result suggests that the downstream signaling cascade of TNFα, TRAF3 and GADD45A in the HCC cells may be merged to the same signaling pathway, such as the JNK signaling pathway (Georgopoulos et al., 2006; Kim et al., 2010; Liebermann et al., 2011). Subsequently, knockdown of either TNFα, TRAF3 or GADD45A will inhibit the JNK signaling pathway and abolish the combination treatment-enhanced apoptosis. It is well-known that JNK plays a critical role in mediating both extrinsic and intrinsic apoptosis (Dhanasekaran and Reddy, 2008); whether it mediates the combination treatment-enhanced apoptosis upon the elevation of TNFα, TRAF3 and GADD45A in the HCC cells deserves further investigation.

Interestingly, TNFα, is also an important inflammatory cytokine, it is a central mediator of inflammation that promotes tumor growth (Li and Jian, 2018). In a study, anti-TNF-α antibodies (infliximab and etanercept) reduces HCC cell viability via antibody-dependent cell-mediated cytotoxicity and complement-dependent cytotoxic effects (Li and Jian, 2018). TRAF3 also regulates cell survival and cytokine production (Hu et al., 2016). Furthermore, both TNFα and TRAF3 activate NFκB (Karin et al., 2004). The activation of NFκB by TNF *a* increases the transcriptions of pro-inflammatory genes such as A20, cIAP-1, cIAP-2, Bcl-xL, XIAP, and IEX-1L (Karin et al., 2004). These target genes also have anti-apoptotic properties (Karin et al., 2004). Furthermore, NFκB induces the antioxidant manganese superoxide dismutase (MnSOD) that has anti-apoptotic effect (Kamata et al., 2005). In our study, we have examined the activity of NFκB after the treatments. The combination treatment increases the activity of NFκB in the HCC cells although it does not reach statistical significance, whereas the treatment slightly but significantly increases the NFκB activity in the tumor tissues. Nevertheless, since combination treatments have more potent growth inhibitory effects than the mono-treatments, we suggest that the activation of NFκB does not significantly affect the cancer growth under the combination treatment.

In face of the dual roles of TNFα and TRAF3 in inducing apoptosis and activating NFκB, blocking the NFκB activity may further enhance the therapeutic efficacy of the combination treatment. Indeed, many naturally occurring and synthetic compounds are able to sensitize cancer cells to TNF-induced cell death by inhibiting NF-κB activity (Zhang et al., 2004; Wang et al., 2006). For example, Akt inhibitor enhances TNF-induced cell death because Akt contributes to TNF-induced NFκB activation (Wang et al., 2007). TNF-induced apoptosis in cancer cells can also be increased by overexpressing the inhibitor of NFκB such as IκB, or by co-administration of selective NFκB inhibitor (Wang et al., 1999; Orlowski and Baldwin, 2002).

In this study, we have combined artesunate and wogonin to treat HCC, which exerts a synergistic effect and a more potent anti-HCC effect than sorafenib does. At the dosages of artesunate and wogonin we used for the animal study, no apparent toxicity is observed. The results suggest that the combination treatment is safe. Artesunate is an approved frontline treatment for severe malaria and wogonin is a natural herbal compound isolated from *Scutellaria baicalensis* which is commonly used in Chinese medicine, the combination of artesunate and wogonin is less likely to have toxicity although comprehensive toxicity tests have to be done to ascertain its safety for clinical practice.

## Conclusions

Our study clearly demonstrates the combination of artesunate and wogonin exhibits synergistic anti-HCC effect and have delineated the underlying mechanism of action. Our data support the further development of this combination as alternative therapeutics for HCC management.

## Data Availability

The original contributions presented in the study are included in the article/Supplementary Material, further inquiries can be directed to the corresponding authors.

## References

[B1] Cabral L. K. D. Tiribelli C. Sukowati C. H. C. (2020). Sorafenib resistance in hepatocellular carcinoma: the relevance of genetic heterogeneity. Cancers 12 (6), 1576. 10.3390/cancers12061576 PMC7352671 32549224

[B2] Chao C. T. (2010). Drug combination studies and their synergy quantification using the Chou-Talalay method. Cancer Res. 70 (2), 440–446. 10.1158/0008-5472.CAN-09-1947 20068163

[B3] Chow S. E. Chen Y. W. Liang C. A. Huang Y. K. Wang J. S. (2012). Wogonin induces cross‐regulation between autophagy and apoptosis via a variety of Akt pathway in human nasopharyngeal carcinoma cells. J. Cel. Biochem. 113 (11), 3476–3485. 10.1002/jcb.24224 22689083

[B4] Dhanasekaran D. N. Reddy E. P. (2008). JNK signaling in apoptosis. Oncogene 27, 6245–6251. 10.1038/onc.2008.301 18931691 PMC3063296

[B5] Ding J. Qin D. Zhang Y. Li Q. Li Y. Li J. (2020). SMAC mimetic birinapant inhibits hepatocellular carcinoma growth by activating the cIAP1/TRAF3 signaling pathway. Mol. Med. Rep. 21 (3), 1251–1257. 10.3892/mmr.2020.10908 31922244 PMC7002966

[B6] Georgopoulos N. T. Steele L. P. Thomson M. J. Selby P. J. Southgate J. Trejdosiewicz L. K. (2006). A novel mechanism of CD40-induced apoptosis of carcinoma cells involving TRAF3 and JNK/AP-1 activation. Cell Death Differ 13 (10), 1789–1801. 10.1038/sj.cdd.4401859 16429118

[B7] Greten T. F. (2019). Gender disparity in HCC: is it the fat and not the sex?. J. Exp. Med. 216 (5), 1014–1015. 10.1084/jem.20190441 31000682 PMC6504214

[B8] Hong M. Almutairi M. M. Li S. Li J. (2020). Wogonin inhibits cell cycle progression by activating the glycogen synthase kinase-3 beta in hepatocellular carcinoma. Phytomedicine 68, 153174. 10.1016/j.phymed.2020.153174 31991293

[B9] Hong M. Cheng H. Song L. Wang W. Wang Q. Xu D. (2018). Wogonin suppresses the activity of matrix metalloproteinase-9 and inhibits migration and invasion in human hepatocellular carcinoma. Molecules 23 (2), 384. 10.3390/molecules23020384 PMC6017513 29439451

[B10] Hou J. Wang D. Zhang R. Wang H. (2008). Experimental therapy of hepatoma with artemisinin and its derivatives: *in vitro* and *in vivo* activity, chemosensitization, and mechanisms of action. Clin. Cancer Res. 14 (17), 5519–5530. 10.1158/1078-0432.ccr-08-0197 18765544

[B11] Hu J. Zhu X.-H. Zhang X.-J. Wang P.-X. Zhang R. Zhang P. (2016). Targeting TRAF3 signaling protects against hepatic ischemia/reperfusions injury. J. Hepatol. 64 (1), 146–159. 10.1016/j.jhep.2015.08.021 26334576

[B12] Jiang F. Zhou J. Y. Zhang D. Liu M. H. Chen Y. G. (2018). Artesunate induces apoptosis and autophagy in HCT116 colon cancer cells, and autophagy inhibition enhances the artesunate–induced apoptosis. Int. J. Mol. Med. 42 (3), 1295–1304. 10.3892/ijmm.2018.3712 29901098 PMC6089754

[B13] Kamata H. Honda S. I Maeda S. Chang L. Hirata H. Karin M. (2005). Reactive oxygen species promote tnfα-induced death and sustained JNK activation by inhibiting MAP kinase phosphatases. Cell 120 (5), 649–661. 10.1016/j.cell.2004.12.041 15766528

[B14] Karin M. Yamamoto Y. Wang Q. M. (2004). The IKK NF-κB system: a treasure trove for drug development. Nat. Rev. Drug Discov. 3 (1), 17–26. 10.1038/nrd1279 14708018

[B15] Kim J. J. Lee S. B. Park J. K. Yoo Y. D. (2010). TNF-α-induced ROS production triggering apoptosis is directly linked to Romo1 and Bcl-XL. Cel Death Differ 17 (9), 1420–1434. 10.1038/cdd.2010.19 20203691

[B16] Li H. Xu K. Pian G. Sun S. (2019). Artesunate and sorafenib: combinatorial inhibition of liver cancer cell growth. Oncol. Lett. 18 (5), 4735–4743. 10.3892/ol.2019.10810 31611983 PMC6781774

[B17] Li J. Zhou B. (2010). Biological actions of artemisinin: insights from medicinal chemistry studies. Molecules 15 (3), 1378–1397. 10.3390/molecules15031378 20335987 PMC6257283

[B18] Li W. Jian Y. B. (2018). Antitumor necrosis factor-α antibodies as a noveltherapy for hepatocellular carcinoma. Exp. Ther. Med. 16 (2), 529–536. 10.3892/etm.2018.6235 30116311 PMC6090380

[B19] Liebermann D. A. Tront J. S. Sha X. Mukherjee K. Mohamed-Hadley A. Hoffman B. (2011). Gadd45 stress sensors in malignancy and leukemia. Crit. Rev. Oncog 16 (1-2), 129–140. 10.1615/critrevoncog.v16.i1-2.120 22150313 PMC3268054

[B20] Lim E. J. Torresi J. (2021). Prevention of hepatitis C virus infection and liver cancer. Recent Results Cancer Res. 217, 107–140. 10.1007/978-3-030-57362-1_6 33200364

[B21] Morris C. A. Duparc S. Borghini-Fuhrer I. Jung D. Shin C.-S. Fleckenstein L. (2011). Review of the clinical pharmacokinetics of artesunate and its active metabolite dihydroartemisinin following intravenous, intramuscular, oral or rectal administration. Malar. J. 10, 263. 10.1186/1475-2875-10-263 21914160 PMC3180444

[B22] Natri H. M. Wilson M. A. Buetow K. H. (2019). Distinct molecular etiologies of male and female hepatocellular carcinoma. BMC Cancer 19 (1), 951. 10.1186/s12885-019-6167-2 31615477 PMC6794913

[B23] Orlowski R. Z. Baldwin A. S. Jr (2002). NF-κB as a therapeutic target in cancer. Trends Mol. Med. 8, 385–389. 10.1016/s1471-4914(02)02375-4 12127724

[B24] Palmer A. C. Sorger P. K. (2017). Combination cancer therapy can confer benefit via patient-to-patient variability without drug additivity or synergy. Cell 171 (7), 1678–1691. 10.1016/j.cell.2017.11.009 29245013 PMC5741091

[B25] Reiss K. A. Yu S. Mamtani R. Mehta R. D’Addeo K. Wileyto E. P. (2017). Starting dose of sorafenib for the treatment of hepatocellular carcinoma: a retrospective, Multi-Institutional Study. Jco 35 (31), 3575–3581. 10.1200/jco.2017.73.8245 PMC5662845 28872925

[B26] Ringehan M. McKeating J. A. Protzer U. (2017). Viral hepatitis and liver cancer. Phil. Trans. R. Soc. B 372 (1732), 20160274. 10.1098/rstb.2016.0274 28893941 PMC5597741

[B27] Rong L.-W. Wang R.-X. Zheng X.-L. Feng X.-Q. Zhang L. Zhang L. (2017). Combination of wogonin and sorafenib effectively kills human hepatocellular carcinoma cells through apoptosis potentiation and autophagy inhibition. Oncol. Lett. 13 (6), 5028–5034. 10.3892/ol.2017.6059 28599504 PMC5453111

[B28] Tong T. Ji J. Jin S. Li X. Fan W. Song Y. (2005). Gadd45a expression induces Bim dissociation from the cytoskeleton and translocation to mitochondria. Mcb 25 (11), 4488–4500. 10.1128/mcb.25.11.4488-4500.2005 15899854 PMC1140626

[B29] Vandewynckel Y. P. Laukens D. Geerts A. Vanhove C. Descamps B. Colle I. (2014). Therapeutic effects of artesunate in hepatocellular carcinoma: repurposing an ancient antimalarial agent. Eur. J. Gastroenterol. Hepatol. 26 (8), 861–870. 10.1097/meg.0000000000000066 24987823

[B30] Villanueva A. Llovet J. M. (2012). Second-line therapies in hepatocellular carcinoma: emergence of resistance to sorafenib.. Clin. Cancer Res. 18 (7), 1824–1826. 10.1158/1078-0432.ccr-12-0151 22355010 PMC3336075

[B31] Wang C. Y. Cusack J. C. Liu R. Baldwin A. S. (1999). Control of inducible chemoresistance: enhanced anti-tumor therapy through increased apoptosis by inhibition of NF-κB. Nat. Med. 5, 412–417. 10.1038/7410 10202930

[B32] Wang X. Chen W. Lin Y. (2007). Sensitization of TNF-induced cytotoxicity in lung cancer cells by concurrent suppression of the NF-κB and Akt pathways. Biochem. Biophysical Res. Commun. 355 (3), 807–812. 10.1016/j.bbrc.2007.02.030 17316570

[B33] Wang X. Ju W. Renouard J. Aden J. Belinsky S. A. Lin Y. (2006). 17-Allylamino-17-Demethoxygeldanamycin synergistically potentiates tumor necrosis factor-induced lung cancer cell death by blocking the nuclear factor-κb pathway. Cancer Res. 66 (2), 1089–1095. 10.1158/0008-5472.can-05-2698 16424045

[B34] Wang X. Lin Y. (2008). Tumor necrosis factor and cancer, buddies or foes?. Acta Pharmacol. Sin 29 (11), 1275–1288. 10.1111/j.1745-7254.2008.00889.x 18954521 PMC2631033

[B35] Wang X. (2001). The expanding role of mitochondria in apoptosis. Genes Dev. 15 (22), 2922–2933. 11711427

[B36] Yang J. D. Hainaut P. Gores G. J. Amadou A. Plymoth A. Roberts L. R. (2019). A global view of hepatocellular carcinoma: trends, risk, prevention and management. Nat. Rev. Gastroenterol. Hepatol. 16 (10), 589–604. 10.1038/s41575-019-0186-y 31439937 PMC6813818

[B37] Zhang S. Lin Z. N. Yang C. F. Shi X. Ong C. N. Shen H. M. (2004). Suppressed NF- B and sustained JNK activation contribute to the sensitization effect of parthenolide to TNF- -induced apoptosis in human cancer cells. Carcinogenesis 25 (11), 2191–2199. 10.1093/carcin/bgh234 15256485

[B38] Zhu J. Jin M. Wang J. Zhang H. Wu Y. Li D. (2018). TNFα induces Ca2+ influx to accelerate extrinsic apoptosis in hepatocellular carcinoma cells. J. Exp. Clin. Cancer Res. 37 (1), 43. 10.1186/s13046-018-0714-6 29506556 PMC5838867

