# Supplementary figures and images for: Combination of Wogonin and Artesunate Exhibits Synergistic anti-Hepatocellular Carcinoma Effect by Increasing DNA-Damage-Inducible Alpha, Tumor Necrosis Factor α and Tumor Necrosis Factor Receptor-Associated Factor 3-mediated Apoptosis
Source: Front Pharmacol. 2021 May 5;12:657080. doi: 10.3389/fphar.2021.657080 (PMC8131852; doi:10.3389/fphar.2021.657080)

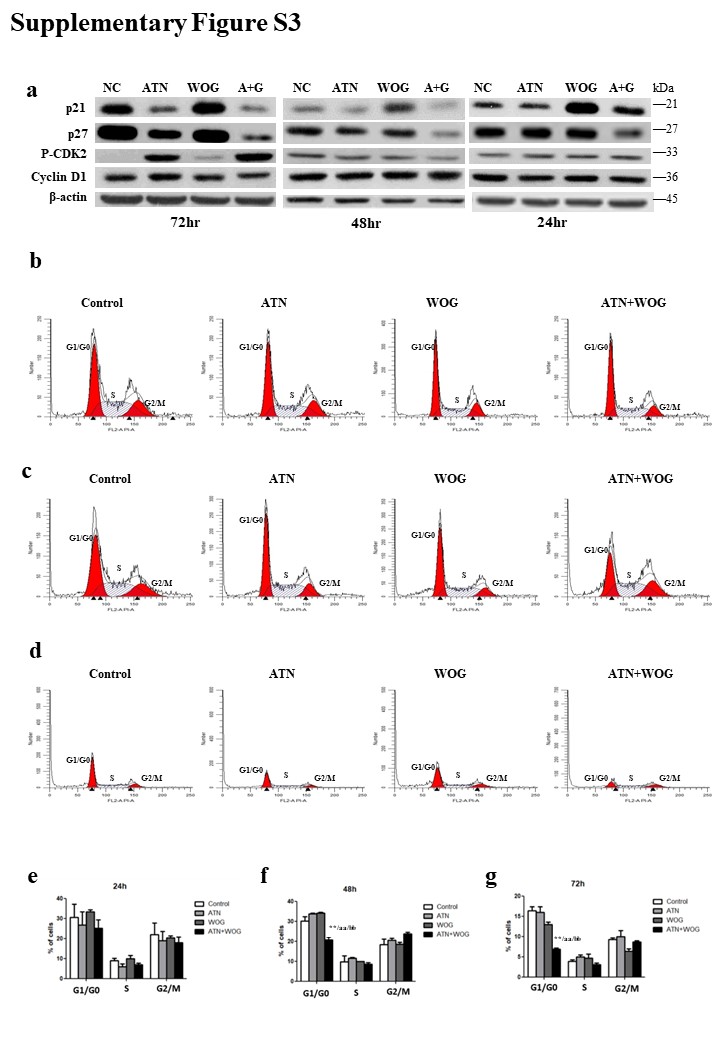

Supplement: Supplementary file 1 [file Image3.JPEG]

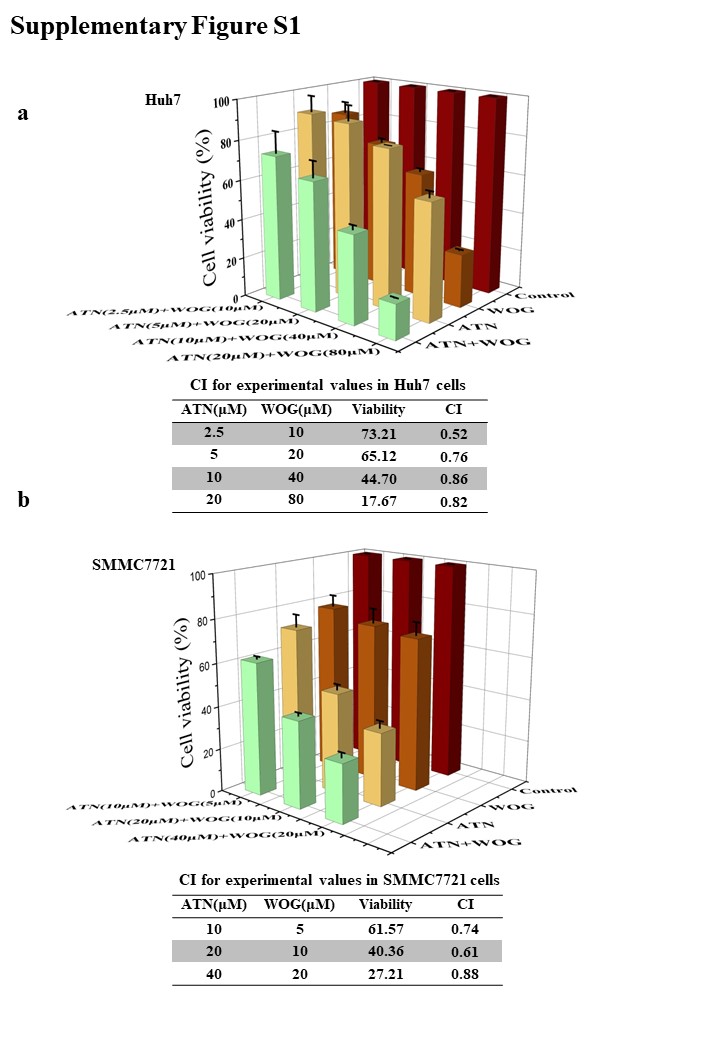

Supplement: Supplementary file 2 [file Image1.JPEG]

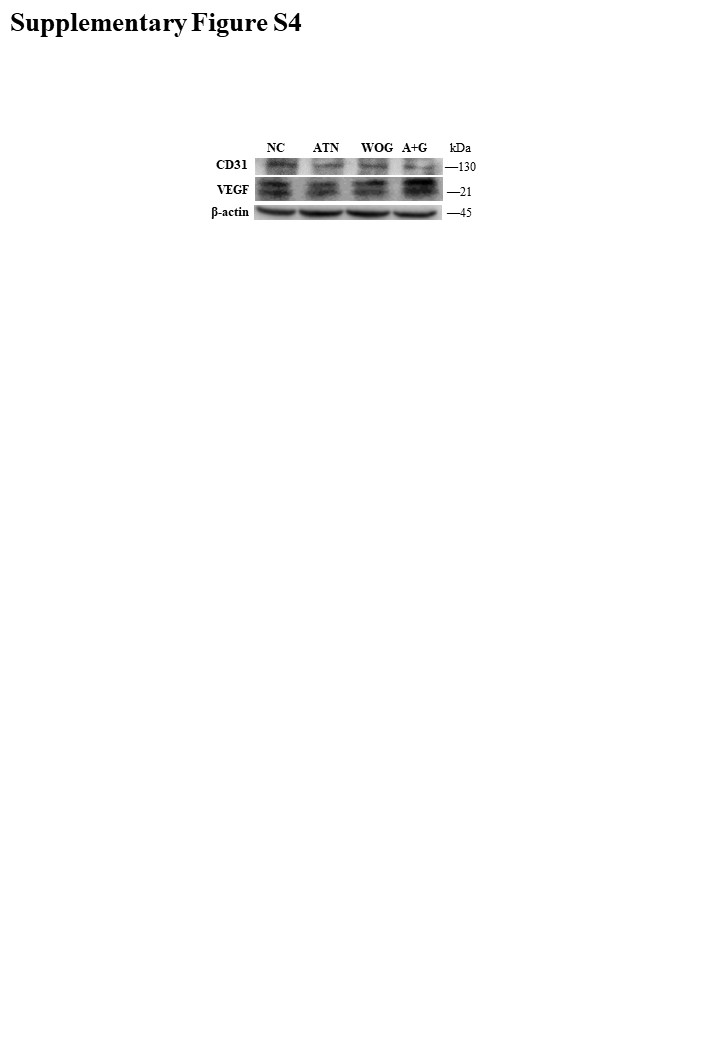

Supplement: Supplementary file 3 [file Image4.JPEG]

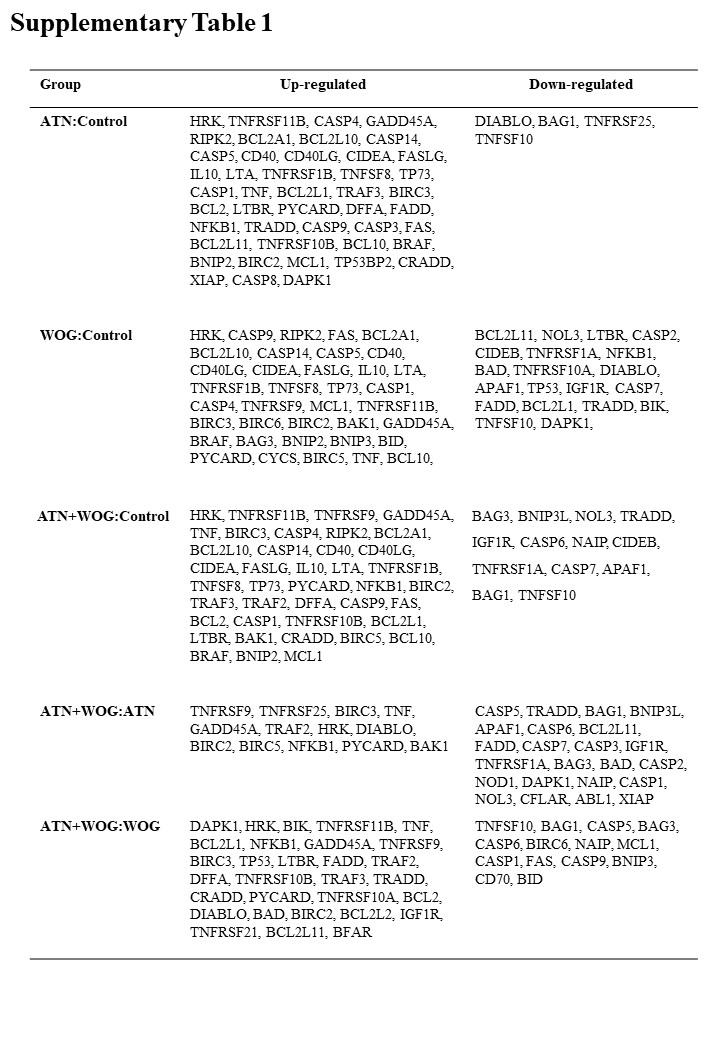

Supplement: Supplementary file 4 [file Image7.JPEG]

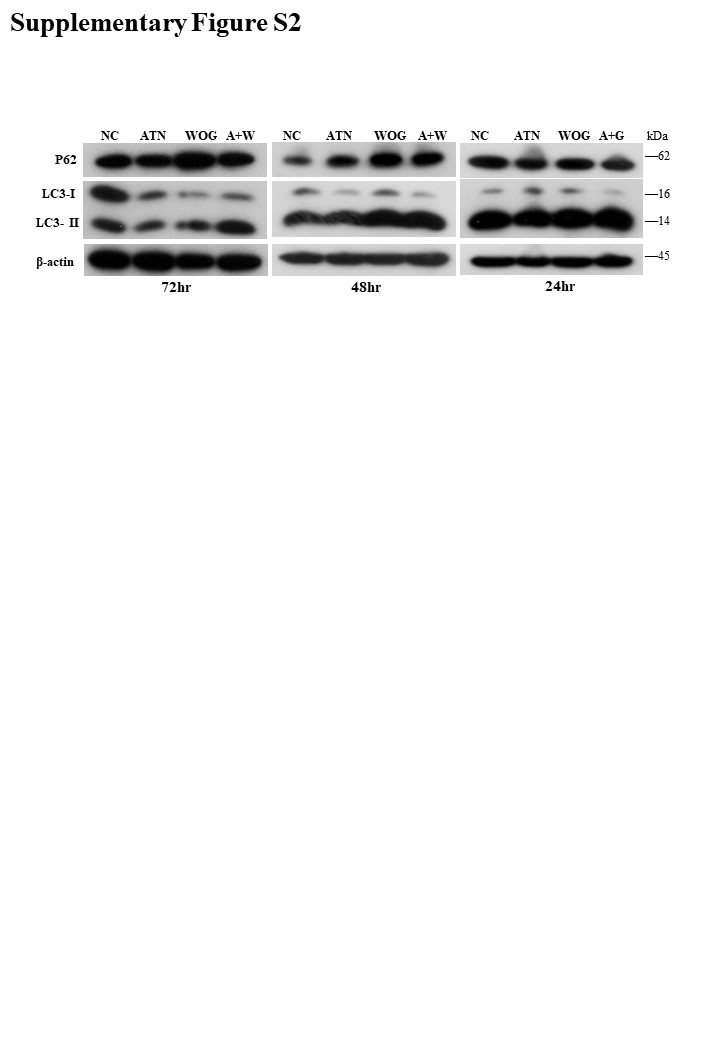

Supplement: Supplementary file 5 [file Image2.JPEG]

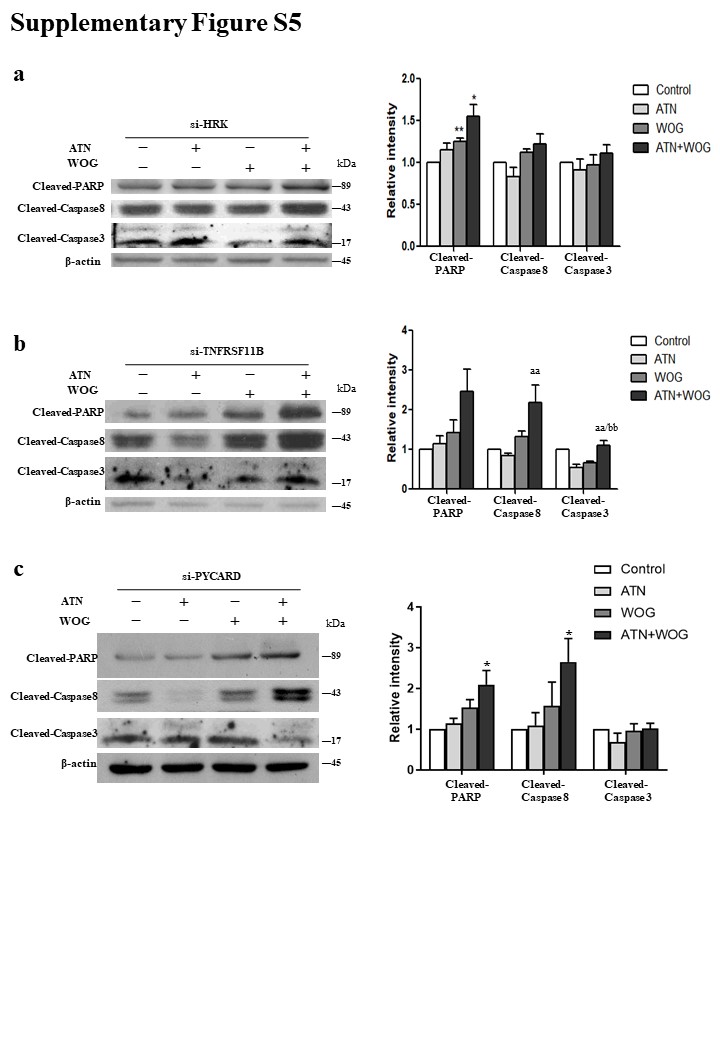

Supplement: Supplementary file 6 [file Image5.JPEG]

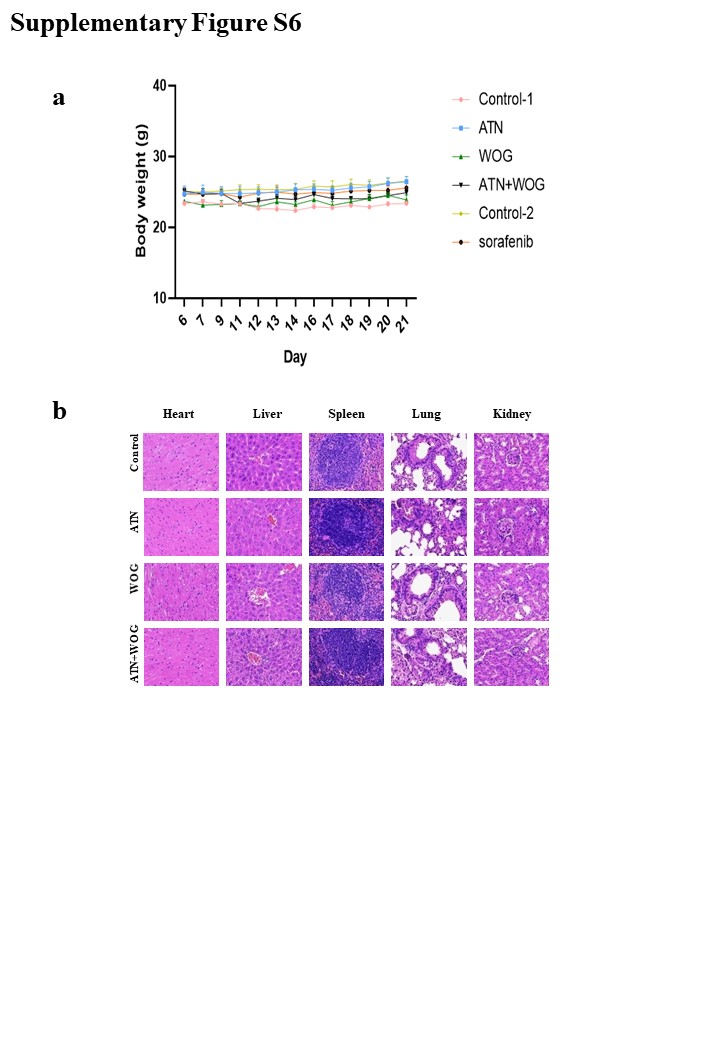

Supplement: Supplementary file 7 [file Image6.JPEG]
